# Fostering a Reflecting Processing of the Academic Crisis: The Effectiveness of Group Counselling for Underachieving University Students

**DOI:** 10.3390/healthcare14121776

**Published:** 2026-06-19

**Authors:** Giovanna Esposito, Raffaella Passeggia, Anna Cannata, Maria Francesca Freda

**Affiliations:** 1 Department of Humanities, University of Naples Federico II, 80133 Naples, Italy; giovan.esposito@unina.it (G.E.); fmfreda@unina.it (M.F.F.); 2 Servizi per l’Inclusione Attiva e Partecipata degli Studenti Universitari Center, University of Naples Federico II, 80133 Naples, Italy; anna.cannata@unina.it

**Keywords:** university counselling, psychological distress, academic underachievement, mentalization, emotion regulation, academic engagement

## Abstract

**Background:** University counselling services provide essential support for students navigating critical academic phases. These services have proven to be successful in promoting long-term psychological well-being and student retention. **Methods:** In a clinical and health psychology perspective, this study aims to analyze the effectiveness of the Narrative Mediation Path (NMP) counselling groups involving 85 underachieving university students, lagging behind in their studies. The intervention aims at promoting psychological well-being, Reflective Functioning, emotion regulation, and academic engagement in order to improve students’ academic performance and prevent university dropouts. At the beginning and end of counselling the following measures were administered: (a) Clinical Outcomes in Routine Evaluation—Outcome Measure, (b) SInAPSi Academic Engagement Scale, (c) Academic Performance Inventory, (d) Reflective Functioning Questionnaire, (e) Emotion Regulation Questionnaire. **Results:** The results showed that counselling group participation was associated with significant and clinical improvement in all the outcomes considered. Reflective Functioning showed a trend toward improvement, but this change did not reach statistical significance. **Conclusions:** Overall, the present study suggested that group counselling could represent a useful service supporting students’ psychological wellbeing and in facing the difficulties encountered during the academic career.

## 1. Introduction

During last years, academic life has become increasingly stressful for students within higher education, as they face a highly competitive environment that demands constant productivity [1,2,3]. In this perspective, the phenomenon of academic underachievement, defined as a discrepancy between potential or predicted ability and actual academic performance [4,5] (or in other words underachieving students are failing to develop and express their potential), gains relevance. Underachievement psychological correlates have been extensively studied in recent decades, and underachieving students present an increased vulnerability to long term social and psychological and behavioral problems [6]. Research also suggests that the psychological health needs of university students are shifting [7] from positive developmental and informational needs toward more concerning manifestations of psychological problems [8]. This shift has led to an increased need for university counselling centres to move beyond simple informational support and prioritize remedial and preventive interventions [9]. The necessity for such interventions is rooted in the evidence that psychological difficulties are not isolated personal issues but are inextricably linked to institutional outcomes; specifically, psychological problems have a direct negative impact on academic performance, retention, and graduation rates [10,11].

From a Clinical Health Psychology perspective [12], university counselling must therefore be framed as a specialized device for the promotion of psychological wellbeing and the prevention of psychological distress and of academic attrition. The psychological distress is a multidimensional construct that affects an individual’s potential [13] and, in the academic setting, acts as a primary barrier to successful functioning [14]. Specifically, high levels of PD may generate a progressive erosion of academic engagement (AE), namely the quality of the academic experience regarding identification, involvement, participation, and effort [15,16]. AE is a proximal predictor of learning outcomes, a mediator between personal/contextual variables and performance [17]. It also acts as an antidote to early dropout and a resource for academic success [18]. Due to its malleability, AE serves as a catalyst for positive processes [19], yielding both short-term benefits on performance and long-term effects on persistence and well-being [18].

The erosion of AE represents a precursor to academic failure: the inability to regulate distress may lead to “academic freeze”, where students stop attending courses and sitting for examinations, experiencing a block in their academic career. A phenomenon such as this may block academic performance, measured mainly by the accumulation of academic delay, and significantly increase the risk of dropout [20,21].

Therefore, counselling interventions are essential not only for distress reduction but for restoring the student’s sense of agency and authorship over their academic career.

Within this context, two psychological resources play a pivotal role in mediating the student’s response to academic stressors: mentalization and emotion regulation, which are bi-directionally linked to one and the other, both theoretically [22] and empirically in clinical and community samples [23,24,25].

Mentalizing [26] is a core psycho-social competence for individuals facing challenging developmental tasks and crises [27]. Also operationalized as Reflective Functioning (RF) [28], it represents the imaginative ability to interpret one’s own and others’ behaviors as driven by intentional mental states, such as desires, needs, and beliefs [26]. However, highly emotional or insecure environments may undermine this capacity by triggering negative self-representations. Such distressing experiences may often disrupt adaptive psychological processing, shifting it into inflexible, non-mentalizing modes that ultimately result in rigid and stereotypical interpretations of one’s context [29,30,31]. Indeed, restoring mentalization with high-functioning individuals (such as university students) may renovate individuals’ sense of agency, i.e., their sense of being the responsible author of one’s own actions. In addition, mentalization also has an impact on psychological well-being, since mentalization sustains a positive perspective on life, hope, and a sense of mastery [32,33,34]. Similarly, emotion regulation, which is associated with genuine mentalization [23,24,25], plays a positive role in psychological well-being. Emotion regulation involves the processes of monitoring, evaluating, and modifying emotional reactions [35]. It comprises four types of strategies that refer to different modes of managing the emotional response to stimuli: situation selection, situation modification, attention deployment or distraction, cognitive change or cognitive reappraisal, and response modulation or expressive suppression. Among these various strategies that individuals may employ to regulate emotions, cognitive reappraisal and expressive suppression are the most studied. A recent meta-analysis [36] showed that cognitive reappraisal positively correlates with psychological well-being indicators and negative correlations with psychological distress indicators. Conversely, expressive suppression demonstrated negative associations with psychological well-being indicators and positive associations with psychological distress indicators.

In this perspective, genuine mentalization allows the regulation of emotions, and a regulated affect allows for mentalizing [22]. When these capacities are compromised, students may be unable to process the anxieties related to university evaluation and performance, leading to a significant increase in PD. By enhancing mentalizing and emotional regulating abilities, counselling aims to break the cycle between psychological suffering and academic disengagement, thereby promoting persistence and successful degree completion [37,38,39].

Although the connection between psychological well-being, mentalizing and affective regulating ability, and performance is widely acknowledged [22,40], empirical research in the university context remains limited [38,41,42,43], necessitating further investigation into how clinical interventions can simultaneously improve mental health and academic outcomes [44,45].

In this perspective, the Narrative Mediation Path (NMP), a group counselling model, has been developed. NMP stands out as a structured intervention specifically designed to utilize narrative devices to re-activate the core reflective processes and emotion regulation strategies to promote distress reduction, and in turn academic engagement and success.

### 1.1. Research Context

The Narrative Mediation Path (NMP) group counselling is a clinical intervention designed for underachieving university students presenting with academic delay, who represent an increase in the risk of dropping out. Adopting a narrative method [46], the NMP aims to promote reflective competencies of mentalization [47] to support psychological well-being, emotion regulation and enhance academic performance [48]. The NMP assumes that improvements in mentalization influence students’ awareness of their thoughts and feelings about their academic delay, thereby impacting their ability to regulate emotions, their sense of agency and the adoption of goal-oriented actions.

The intervention consists of nine weekly sessions organized into five narrative modes: the metaphorical mode (Sessions 1–2), using proverbs and mottos to foster initial representations of the academic role; the iconographic mode (Sessions 3–4), employing vignettes as projective stimuli to recognize resources and difficulties; the writing mode (Sessions 5–6), involving written accounts of positive, negative, and turning-point episodes to encourage new perspectives; the bodily mode (Session 7), using body to sculpt and to reflect on the academic future; and the agency mode (Sessions 8–9), focusing on action plans to transform reflectivity into goal-oriented behaviors. Throughout this process, the function of the group is crucial as an instrument of sharing, exchange, and reflection that activates a dynamic process of mutual influence, allowing members to modify rigid or unjustified beliefs affecting their performance [48]. The counsellor acts as a reflective mirroring agent who co-constructs meaning, models mentalizing ability, and focuses on the “here and now” to validate emotional experiences [30,38,48]. Recent evidence has confirmed that the NMP successfully improves mentalizing ability, specifically increasing certainty and significantly decreasing uncertainty about mental states, while reducing psychological distress and academic delay in both face-to-face and online settings [38,41].

### 1.2. The Present Study

The general aim of the present study was to evaluate the effectiveness of the Narrative Mediation Path (NMP) group counselling in a sample of underachieving university students, through a naturalistic pre-post design. Specifically, the study extends previous research on the NMP by examining a broader set of psychological and academic outcomes in a naturalistic university counselling context [38,41] combining psychological and academic measures in order to investigate whether the intervention could be associated with an improvement in terms of psychological well-being, academic engagement, mentalization, emotion regulation, and academic performance. More specifically, mentalizing and emotion regulation represent proximal processes; psychological distress is the proximal outcome; engagement represents the intermediate outcome; and academic performance is the distal outcome. Based on the theoretical framework of Clinical Health Psychology, the NMP model, and previous studies [38,41], a significant reduction in psychological distress and its dimensions was expected. Additionally, it was hypothesized that the group counselling would be especially associated with improvement for students with mild-to-moderate levels of distress. Similarly, significant improvements in academic engagement, mentalization, emotion regulation, and academic performance were expected.

## 2. Materials and Methods

### 2.1. Participants

The number of students who participated in the group counselling was N = 112. About 25% dropped; therefore, the study involved a total sample of 85 university students, participating in 13 counselling groups, with a range of 4 to 9 members per group. Participants attended the groups voluntarily and self-referred themselves to the service. In this study, all the participants attended at least 7 sessions. Moreover, all participating students were underachieving, as the percentage of their acquired ECTS at baseline on the expected number of ECTS they should have had considering their academic year was below 50%. Participants’ mean age was 24.34 years (*SD* = 5.90). Regarding gender distribution, the sample comprised 58.8% males and 41.2% females. In terms of relationship status, the majority of the participants reported being single (60.0%), while 36.5% were in a relationship, and a small minority were married or in other arrangements (3.5%). Exploring their living arrangements, most students lived with their families (87.1%), with a smaller portion living with roommates (9.4%) or with a partner (2.4%). The sample was relatively balanced regarding daily commute, with 51.4% identifying as commuters and 48.6% as non-commuters. Finally, concerning their academic background, more than half of the participants were enrolled in Science, Technology, Engineering, and Mathematics (STEM) degree courses (53.6%), followed by students in Social Sciences and Humanities (SSH; 31.0%), and Healthcare programs (15.5%). Finally, regarding their academic progress, a considerable portion of the sample (41.2%) consisted of *fuoricorso* students (i.e., students enrolled beyond the regular duration of their degree program).

### 2.2. Measures

#### 2.2.1. Clinical Outcomes Routine Evaluation—Outcome Measure

The CORE-OM [49,50] is the gold standard for psychological distress evaluation. In its version without the risk scale, it comprises 28 items covering the domains of subjective well-being (e.g., ‘I have felt optimistic about my future’), problems and symptoms (e.g., ‘Tension and anxiety have prevented me doing important things’), and functioning, which includes items on general functioning, close relationships, social relationships (e.g., ‘I have felt able to cope when things go wrong’). One half of the items focus on low intensity problems (e.g., ‘I feel anxious or nervous’) and the other focus on high-intensity problems (e.g., ‘I feel panic or terror’). Items are scored from 0 to 4 (anchored not at all, only occasionally, sometimes, often and all or most of the time) and relate to the preceding week. In the present study the CORE-OM was employed in its version excluding the risk scale, which in the Italian validation [51] has excellent reliability (*α* = 0.93). In the current sample, the reliability was excellent for the total scale (*ω* = 0.92), with subscale reliabilities ranging from adequate (*ω* = 0.63 for well-being) to excellent (*ω* = 0.89 for symptoms).

The CORE-OM allows us to obtain a clinical score (cut-off = 10) and to evaluate severity levels as follows: 0–4 psychological health; 5–9 low distress; 10–14 mild distress; 15–19 moderate distress; 20–24 moderate-to-severe distress; above 25 severe distress.

#### 2.2.2. SInAPSi Academic Engagement Scale

In order to measure the students’ academic engagement as a dynamic and contextual process, the SInAPSi Academic Engagement Scale [19] was administered. The SAES operationalizes an academic engagement (AE) model for university students based on six dimensions and the 29 items (on a 5-point Likert scale) are organized into 6 scales, corresponding to each of the six dimensions of the AE model: (1) perception of the capability to persist in the university choice, which refers to the awareness of the encountered (or to be encountered) difficulties and of the resources needed to overcome them; in this perspective, this dimension is strictly connected to the intention to drop out (e.g., ‘I’d be better off doing other things than going to university’); (2) value of university and sense of belonging, which refers to the recognition of the worth of the university choice, of the relevance of the choice to enroll in the life project, and the perception of a sense of belonging to the context (e.g., ‘Attending university is a great opportunity for me’); (3) value of university course, which refers to the recognition of the relevance of the chosen academic course for the professional future; this dimension is connected with the belief that the chosen academic course represents an opportunity, a source of interest and a chance for personal growth (e.g., ‘The course of study I am attending will help me to achieve my professional goals’); (4) relationship with peers, that to the possibility to create positive and meaningful relationship with other students; this dimension refers also to the possibility to consider the group of peers at university as a relevant relational net, not only for academic purpose (studying together) but also as support source (e.g., ‘I have created meaningful friendships with some of my fellow students’); (5) relationship with professors, that refers to the feeling of being “seen” as a person by the faculty members; it also refers to the perception of availability and interest of the professors, and to be respected by them (e.g., ‘My teachers respect me as a person’); (6) integration between university and relational net, which refers to the balance between academic and private life and the recognition of the need to share with family and friends the enthusiasm for the academic project (e.g., ‘I discuss my university career with my family’).

Reliability was excellent in the current sample with *ω* = 0.82 for the total scale and with a range of *ω* = 0.80–0.91 for the subscales.

#### 2.2.3. Reflective Functioning Questionnaire

The Reflective Functioning Questionnaire (RFQ) [52] is the most used self-report measure for evaluating mentalizing. It was employed to assess students’ RF ability at the beginning and end of counselling. The validated Italian version of the RFQ [53] consists of 8 items organized into two 6-item subscales (with two unique and four shared items), measuring the degrees of uncertainty (RFQ-U) and certainty (RFQ-C) regarding mental states. The RFQ-U subscale reflects hypomentalizing—the tendency to develop simplistic models of internal states, often linked to concrete thinking or psychic equivalence modes [52] (e.g., “People’s thoughts are a mystery to me”). Conversely, the RFQ-C subscale captures hypermentalizing, characterized by the construction of overly complex, rigid mental models that lack empirical support [52] (e.g., “I always know what I feel”). Genuine mentalizing is instead distinguished by the ability to form relatively accurate models of minds while recognizing the opacity of mental states [22,32]. Consistent with the existing literature [53,54,55,56], genuine mentalization is indexed by moderate RFQ-C scores (approaching 1) and low RFQ-U scores (approaching 0). Consequently, successful clinical interventions are expected to yield pre-to-post-test improvements characterized by an increase in certainty and a concurrent decrease in uncertainty scores.

Reliability in the current sample was adequate with *ω* = 0.76 for the total scale; adequate for uncertainty with *ω* = 0.75 and below adequacy for certainty with *ω* = 0.53.

#### 2.2.4. Emotion Regulation Questionnaire

The Emotion Regulation Questionnaire (ERQ) [57,58] is a 10-item self-report questionnaire which consists of two scales corresponding to two different emotion regulation strategies: cognitive reappraisal, which comprises 6 items (e.g., ‘When I want to feel less negative emotion (such as sadness or anger), I change what I’m thinking about.’) and refers to the cognitive change in an individual’s understanding of an emotion-inducing situation or event, thus changing the emotional experience, which occurs in the early stage of the emotional generation process, and expressive suppression, which includes 4 items (e.g., ‘I control my emotions by not expressing them’) and refers to a response-focused strategy that involves continuously inhibiting emotion expression behavior to reduce subjective emotional experience [59]. The 10 items are rated on a 7-point-Likert scale from strongly disagree to strongly agree.

The measure showed good psychometric properties in its Italian adaptation and reliability in the current sample was excellent with *ω* = 0.89 for cognitive reappraisal and adequate with *ω* = 0.72 for expressive suppression.

#### 2.2.5. Academic Performance Inventory

In order to assess the impact of the counselling groups, the Academic Performance Inventory (API) [46] was administered to participants before and at the end of the intervention. The API consists of questions related to the university career of the participants (number of exams taken, European Credits Transfer System ECTS gained, the year of enrollment, the number of exams passed, etc.) (number of exams taken, European Credits Transfer System ECTS gained, the year of enrollment, the number of exams passed, etc.).

### 2.3. Procedures

The counselling groups were held during the academic years 2023–2025 and they were conducted by two different clinical psychologists and psychotherapists specifically trained in the use of the NMP methodology and with over 15 years of experience in the delivery of NMP manualized intervention [46,47,48]. To ensure treatment integrity, the clinicians who deliver the intervention undergo bi-monthly supervisions. Students were informed about the intervention through institutional e-mail and social media. The participation was voluntary and all the students signed an informed consent in accordance with the Italian Privacy and Data Protection Act (No. 196/2003), the ethical principles of the Italian Association of Psychology (AIP) and the Helsinki Declaration. Through informed consent, students agreed to the use of narrative materials and data provided in questionnaires for educational and research purposes. Moreover, the research protocol was approved by the Ethical Committee of the University of Naples Federico II (n. prot. 41/2023).

### 2.4. Data Analysis

Statistical analyses were performed using IBM SPSS Statistics (version 30.0) to evaluate the outcomes of the counselling intervention.

Prior to data analysis, the presence of missing data was considered. Specifically, of the 85 students who participated in at least 7 out of 9 sessions, 4 students’ responses to the pre-test instruments were not collected due to a technical problem. The missing data were handled by pairwise exclusion. First, descriptive statistics, including means, standard deviations, and frequencies, were computed to outline the demographic and academic characteristics of the sample. Moreover, kurtosis and skewness were assessed to ensure normality of the variables. All the variable showed kurtosis and skewness in the accepted ranges (respectively ±3 for kurtosis and ±3 for skewness) [60]. To evaluate whether the counselling intervention was associated with an improvement in terms of academic performance, paired-samples *t*-tests were performed on the number of passed exams and the absolute number of university credits (ECTSs) acquired at pre- and post-test.

Similarly, paired-samples *t*-tests were performed to compare pre- and post-counselling scores across all standardized psychological measures (CORE-OM, SAES, ERQ, and RFQ). Effect sizes (ES) measured with Cohen’s *d* for all the comparisons were also examined, considering a small ES of about 0.20, medium about 0.50, and large 0.80 or higher. Furthermore, beyond statistical significance, the clinical relevance of the psychological distress outcomes was evaluated using the CORE-OM. Particularly, severity levels in pre- and post-test were evaluated following established guidelines [50] that identify 5 levels: psychological health (range 0–4); low and non-clinical distress (5–9); mild (10–14); moderate (15–19); moderate–severe (20–24); severe (25 and above). Finally, a visual inspection of the RFQ scores was conducted to qualitatively explore and better understand the pre-post response patterns and shifts within its specific subscales, beyond the statistical significance, in order to identify trends according to the literature, with certainty increasing and uncertainty decreasing [52].

## 3. Results

### 3.1. Outcome Results

#### 3.1.1. Psychological Distress

**Figure 1:**
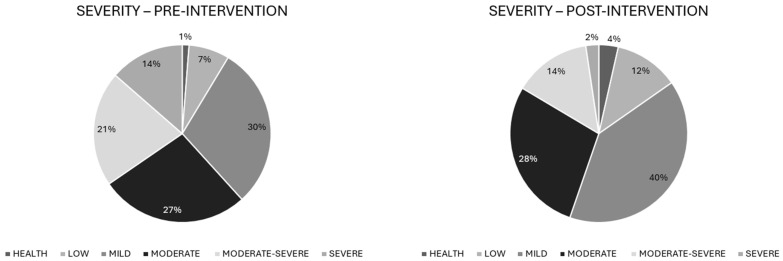
CORE-OM severity levels pre- and post-intervention.

Students reported a highly significant reduction in total psychological distress from pre-counselling (*M* = 16.90, *SD* = 6.01) to post-counselling (*M* = 13.81, *SD* = 5.62), with a medium effect size (*p* < 0.001, *d* = 0.50, medium ES). Significant improvements were also found across all CORE-OM subscales (see Table 1). Beyond mean differences, severity levels pre- and post-intervention were evaluated. At baseline, a large majority of students fell within the clinical range, varying from mild (30%) to severe (14%) distress (see Figure 1). At the end of the intervention, severe distress dropped from 14% to 2% and moderate–severe distress decreased from 21% to 14%. The non-clinical categories increased from an overall 8% to 16%, as well as the mild level, which increased from 30% to 40%, while moderated level stayed fairly stable.

#### 3.1.2. Academic Engagement

Overall academic engagement improved significantly (*p* < 0.001, *d* = 0.43, medium ES) over the course of the intervention (see Table 1). When analyzing specific dimensions, students reported significant gains in university value (*p* < 0.001, *d* = 0.57, medium ES), course value (*p* = 0.001, *d* = 0.40, medium-small ES), and relationships with peers (*p* = 0.007, *d* = 0.31, small ES). Conversely, scores related to capability, integration, and relationships with professors, although they showed an improvement, did not change in a statistically significant manner.

#### 3.1.3. Reflective Functioning

**Figure 2:**
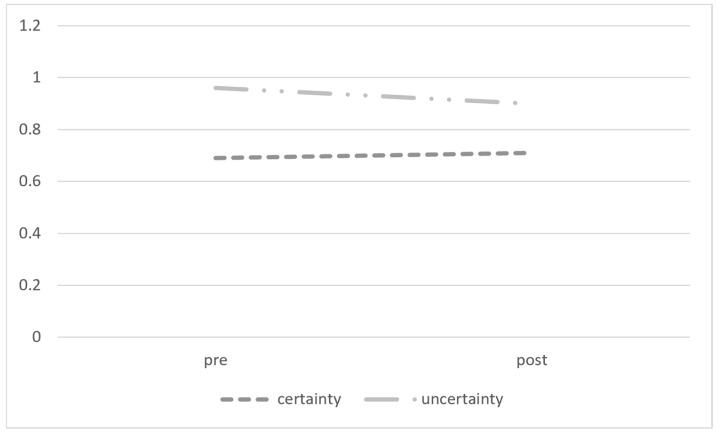
Visual inspection of pre- and post-intervention scores for RFQ dimensions.

Regarding mentalization capabilities, the total Reflective Functioning score did not show any statistically significant change from pre-counselling to post-counselling (*p* = 0.499, *d* = 0.08, small ES). Similarly, its subscales yielded no significant differences: certainty (*p* = 0.811) and uncertainty (*p* = 0.437). To further explore these outcomes and to explore whether the trends were in line with the literature, a visual inspection was conducted specifically for the certainty and uncertainty subscales via line plots (see Figure 2). The graphical representation showed a tendency to increase for certainty and to decrease for uncertainty, as expected, although these variations were not statistically significant.

#### 3.1.4. Emotion Regulation

Regarding emotion regulation strategies, there was a significant increase in the use of cognitive reappraisal (*p* = 0.018, *d* = 0.27, small ES). Additionally, a decrease, although not significant, in the use of expressive suppression was observed (*p* = 0.053, *d* = 0.22, small ES), suggesting an overall shift toward more adaptive emotional regulation profiles.

### 3.2. Impact Results

#### Academic Performance

To evaluate whether the counselling fostered an improvement in terms of academic performance, objective academic metrics were analyzed. Results indicated a significant increase in the absolute number of acquired credits (ECTSs) from pre-intervention to post-intervention (*p* = 0.035, *d* = 0.23, small ES). Accordingly, the total number of passed exams also increased significantly during the period of care (*p* = 0.001, *d* = 0.39, small ES), with students successfully completing roughly one additional exam on average (see Table 1).

## 4. Discussion

The findings of this study provided a picture of the psychological and academic changes occurring during a university group counselling intervention with regard to pre-post measurements.

The most striking result was the significant reduction in global psychological distress. This improvement was also observed across all subscales of the CORE-OM and could indicate that the students’ overall sense of well-being and their ability to maintain social functioning improved during the intervention. The relevant shift from more severe to more functional distress levels in association with the intervention may suggest that the counselling may have provided a contained space where acute developmental crises could be processed, allowing students to regain a sense of agency that had been compromised by their initial distress. Contrary to expectations, the group counselling was associated with a significant reduction in severe distress levels. These findings suggest that, unlike what previous studies suggested on stepped care models [61,62,63], counselling interventions could be functionally used as a first containment space even by students referring to the psychological service and reporting severe distress levels, who could show, however, significant benefit from low-intensity intervention.

The distress reduction could be a driver behind the improvement in academic performance, given the link between psychological well-being and academic outcomes. The data showing a significant increase in the number of exams passed and credits (ECTSs) earned could suggest that the reduction in psychological distress acted as a catalyst for academic mobilization and against underachievement [64]. Clinically, this could be interpreted as the resolution of an academic block: when students are overwhelmed by psychological distress, their cognitive resources may be entirely consumed by emotion regulation, leaving limited capacity for study and concentration. With the alleviation of this emotional burden associated with the intervention, it may be possible to suggest that the counselling intervention likely freed up these cognitive resources, facilitating a successful reinvestment in academic tasks [65]. This success may subsequently create a positive feedback loop: as students resume passing exams, their academic self-confidence is reinforced, further diminishing the avoidant behaviors that typically characterize academic blocks [66,67]. This synergy between emotional relief and academic achievement may suggest that university counselling could be a crucial institutional tool for student retention and success [68].

In line with this interpretation are the changes in academic engagement; the significant increase in university and course value suggests a shift in students’ perception of their academic path, reconceptualizing it as a meaningful investment rather than a stressful environment. Furthermore, the improvement in peer relationships points to a reduction in the social withdrawal often associated with academic blocks, suggesting that improved emotional balance fosters a more supportive social network within the university [69].

The lack of significant change in relationships with faculty and integration scales is an interesting result. Regarding the integration dimension, its non-significant improvement may be interpreted in light of the scale’s nature. In fact, this dimension refers to the link between their academic life and their social network, which also includes the family (especially parents), which generally constitutes a vertical relationship [70], similarly to the relationship with professors. Sharing information with parents could be a source of stress and anxiety, given that in most cases parents are the ones paying for the education. In this perspective, the disclosure of university-related information could depend on the quality of the information itself; therefore, students may be more willing to report positive information to their parents, rather than negative information, especially for first-generation students [71].

Concerning the relationship with professors, given that NMP is based on narrative inputs that very frequently engage students into taking the perspective of their professors, the fact that this dimension did not improve significantly is unexpected. On the one hand, might indicate that while students improved their motivational value and peer support, their perception of the faculty could be less susceptible to change through a brief intervention and therefore require more time. On the other hand, the group format likely provided direct opportunities to engage in concrete functional peer relationships, fostering a deeper development of the specific AE dimension of peer relationships, while, although often at the center of the students’ narratives, the relationship with faculty may have been addressed mostly narratively.

With regard to the mentalization, the findings showed that certainty increased and uncertainty decreased as expected [55], although not statistically significant. On the one hand, these results seem to support that Reflective Functioning, as measured by the RFQ, could function more as a trait rather than a state-dependent function [72,73]; therefore, achieving substantial and measurable modifications in Reflective Functioning likely requires longer or more intensive interventions. In this perspective, to evaluate this hypothesis and confirm the assumption that NMP works on psychological distress and academic engagement through the stimulation of mentalizing capacities, follow-up assessments and a combination with different measures for mentalization need to be performed (also considering the low reliability of the certainty scale, which calls for caution in interpreting these results and their generalizability). On the other hand, these results, alongside the clinical improvement and academic success associated with the intervention, could be interpreted in conjunction with the emotion regulation result, particularly with cognitive reappraisal, given their established relationship. From this perspective, the significant improvement in cognitive reappraisal regulation may support the hypothesis that the group counselling could have represented a context, an ‘arena’ in which to experiment with the processing of emotions and their link to psychological processes capable of relieving distress and mobilizing resources for academic tasks. The significant increase in cognitive reappraisal associated with the intervention suggests that students could have learnt to reframe stressful situations more adaptively [74], which likely contributed to their academic progression. However, the marginal change in suppression indicates that the tendency to hide emotions remained somewhat stable. Nevertheless, this result aligns with previous research indicating that suppression is not necessarily a sign of dysfunction in emotion regulation, but a useful strategy that could act as a protective factor against severe distress; thus, it could help to manage the distress, especially in specific situations and notwithstanding the predominant use of cognitive reappraisal [75]. Accordingly, this finding may also suggest that suppression operates as a lingering defensive mechanism, remaining active to protect against psychological distress [76,77,78].

The counselling is significantly associated with performance improvement and reduced suffering for students referred to the service. This could imply that short-term university counselling, such as the NMP, could act as a clinical bridge [79] for developmental crisis management and functional recovery, providing the tools to overcome the risk of academic block, even if a structural reorganization of mentalizing and emotional regulating capacities would require more intensive or prolonged therapeutic work.

## 5. Conclusions

Overall, the findings provide promising preliminary evidence that participation in NMP group counselling is associated with improvements in psychological distress, academic engagement, emotion regulation, and academic performance among underachieving university students. Future research using controlled designs, follow-up assessments, and multi-site samples is needed to confirm these findings and clarify the mechanisms of change. The intervention participation was associated with a significant reduction in psychological distress and a parallel improvement in academic performance, as evidenced by the increase in examinations passed and credits acquired from the pre- to the post-intervention phase. These findings suggest that structured group counselling models such as the NMP may be a promising support strategy for underachieving students, although further controlled and multi-site studies are needed before broader conclusions can be drawn. However, the non-significant change in Reflective Functioning scores throughout the intervention could indicate that while short-term counselling is associated with symptomatic manifestations reduction and functional recovery, university counselling could also be able to activate this capacity and create the optimal conditions for a structural change, which nonetheless requires more time to unfold. This highlights the dual nature of university counselling: it could serve as a crucial device to secure academic persistence and mental well-being in the short term in overcoming developmental crisis, but, on the other hand, the students who refer to the counselling services are often involved in more than a developmental crisis, and their psychological suffering, although related to the university context, could underlie disfunctions and difficulties that spread and cover more life contexts.

Despite these promising results, several limitations must be acknowledged. First, the absence of a control group limits the ability to definitively attribute the observed changes solely to the NMP intervention rather than to spontaneous remission or other external factors, as highlighted also by previous studies with different samples [38,41,80]. In addition, although the intervention is manualized, no adherence measure was used to ensure treatment integrity, only bi-monthly supervision. Second, the sample size may limit the generalizability of the findings to the specific local culture or to the broader university counselling population. Moreover, the analysis was not performed considering the nested nature of the data. Third, the study relied exclusively on pre- and post-test data; without a longitudinal follow-up, it is impossible to determine the long-term stability of the academic gains, especially considering that the underlying mentalizing structures remained unchanged. Moreover, this study did not consider the contextual factors that contribute to underachievement. Future studies should include measures that take into account the effect of contextual and academic factors. In addition, it should be noted that no formal adjustment for multiple comparisons was applied to the analyses of the 17 subscales. While this choice was preferred to preserve the statistical power of the design given the small sample size and to avoid inflation of Type II errors, results close to the significance threshold should be interpreted with caution and warrant replication in future studies. Finally, the reliance on self-report measures for psychological variables introduces the possibility of social desirability bias. Future research should incorporate randomized control groups and longitudinal designs to further investigate whether the observed functional improvements can eventually lead to structural changes in mentalizing over time.

## Figures and Tables

**Table 1:** Means, *p*-values and effect size for exams, ECTSs, CORE-OM, SAES, RFQ, and ERQ at pre- and post-test.

	Pre-Test Mean (SD)	Post-Test Mean (SD)	*t* Value	*p* Value	Cohen’s *d*
Exams	8.75 (7.12)	9.79 (7.52)	−3.58	0.001	0.39
ECTSs	75.19 (61.84)	79.14 (64.22)	−2.15	0.035	0.29
CORE-OM—functioning	1.72 (0.67)	1.48 (0.60)	3.01	0.004	0.33
CORE-OM—problems and symptoms	2.21 (0.88)	1.72 (0.93)	4.71	<0.001	0.52
CORE-OM—well-being	2.30 (0.87)	1.92 (0.93)	3.29	0.001	0.37
CORE-OM—global	16.9 (6.01)	13.81 (5.62)	4.49	<0.001	0.50
SAES—capability	3.92 (0.84)	3.96 (0.85)	−0.44	0.662	0.05
SAES—university value	3.91 (0.79)	4.18 (0.73)	−5.12	<0.001	0.57
SAES—course value	3.96 (0.87)	4.18 (0.83)	−3.60	0.001	0.40
SAES—integration	3.09 (1.25)	3.12 (1.26)	−0.30	0.765	0.03
SAES—relationship with peers	3.25 (1.06)	3.42 (1.12)	−2.77	0.007	0.31
SAES—relationship with professors	3.12 (0.95)	3.22 (1.00)	−1.30	0.198	0.14
RFQ-C	0.69 (0.55)	0.71 (0.60)	−0.24	0.811	0.03
RFQ-U	0.96 (0.52)	0.90 (0.59)	0.78	0.437	0.09
RFQ	−0.27 (0.90)	−0.20 (1.04)	−0.68	0.499	0.08
ERQ—cognitive reappraisal	4.47 (1.30)	4.77 (1.25)	−2.42	0.018	0.27
ERQ—suppression	3.98 (1.33)	3.72 (1.43)	1.96	0.053	0.22

## Data Availability

The data supporting the present article are available from the corresponding author upon reasonable request due to privacy.

## Abbreviations

The following abbreviations are used in this manuscript:

PD	Psychological distress
AE	Academic engagement
RF	Reflective functioning
ER	Emotion regulation
